# A method of studying the course of myocardial ischemia and reperfusion in rats in vivo

**DOI:** 10.1186/1532-429X-14-S1-P58

**Published:** 2012-02-01

**Authors:** Darach O h-Ici, Thore Dietrich, Daniel Messroghli, Titus Kuehne

**Affiliations:** 1 Cardiovascular Imaging, Department of Congenital Heart Defects and Pediatric Cardiology, Deutsches Herzzentrum Berlin, Berlin, Germany

## Background

The use of MRI for the study of ischemia and its consequences in small animals has been limited by the need for a thoracotomy and operative occlusion of the coronary arteries. The trauma of the surgery may be an important confounder in this open-chest model. The closure of the coronaries with a suture does not allow multiple occlusion-reperfusion cycles, and has limited the study of ischemia/reperfusion in small animals.

The purpose of this study was to develop a “closed chest” model of ischemia/reperfusion, which would allow ischemia and infarction to be studied in real-time while the rat is in the MRI environment.

## Methods

We developed a method of implanting a balloon occluder to the left anterior descending coronary artery. Male Sprague Dawley rats (n=12, weight 367 +/-67 g) were anaesthetized and then intubated. The heart was exposed by an incision between the fourth to fifth rib space. The occluder was then secured loosely to the myocardium with a 6-0 non-absorbable suture. Occlusion and reperfusion of the coronary artery was confirmed by visual inspection (blanching of the left ventricle) and by ECG (ST-segment elevation and normalization) on brief inflation and deflation of the balloon. The tubing of the occluder was then tunneled to the back of the rat and exposed in the intra scapular area. The animals were then allowed to recover from the operation for at least 5 days.

For coronary occlusion and MRI scanning, rats were again anaesthetized, the tubing was connected to a syringe, and animals were placed in the MRI scanner.

## Results

There was a very low mortality rate for the implantation of the coronary occluder. (8.3%). Inflation of the occluder on the MRI table resulted in myocardial ischemia in all animals as documented by ECG, and allowed the effects of ischemia and reperfusion on myocardial edema and function to be studied serially before, during, and after coronary occlusion.

## Conclusions

The use of a pre-implanted balloon occluder allows for studying of the effects of single or repeated myocardial ischemia and reperfusion with MRI in real-time in a closed-chest rat model.

## Funding

This study was funded by the Deutsche Forschungsgesellschaft.

